# Multiple roles of RARRES1 in prostate cancer: Autophagy induction and angiogenesis inhibition

**DOI:** 10.1371/journal.pone.0180344

**Published:** 2017-07-05

**Authors:** Arpita Roy, Malathi Ramalinga, Okjin J. Kim, Juliet Chijioke, Solomon Lynch, Stephen Byers, Deepak Kumar

**Affiliations:** 1 Cancer Research Laboratory, University of the District of Columbia, Washington, DC, United States of America; 2 Lombardi Comprehensive Cancer Center, Georgetown University, Washington, DC, United States of America; 3 JLC-BBRI Nutrition Research lab, North Carolina Central University, Kannapolis, North Carolina, United States of America; 4 Julius L. Chambers Biomedical Biotechnology Research Institute, North Carolina Central University, Durham, North Carolina, United States of America; Wake Forest Baptist Medical Center, UNITED STATES

## Abstract

**Background:**

Prostate cancer (PCa) poses a major health concern in men worldwide. Retinoic Acid Receptor Responder (RARRES1)/ Tazarotene-induced gene-1 (TIG-1) is a putative tumor suppressor gene that exerts its tumor suppressor function via unknown mechanisms. Epigenetic silencing of RARRES1 leads to its loss in several types of cancer, including PCa. Determining the molecular mechanisms that mediate the tumor suppressor role of RARRES1 in PCa is the focus of our study.

**Findings:**

Our data indicates that RARRES1 over expression in PCa cell lines represses mitogen-activated protein kinase (MAPK) activation. RARRES1 expression induces the levels of autophagy-related genes, beclin, ATG3 and increases LC3B-II conversion. A significant induction of SIRT1 along with mTOR inhibition is noted on RARRES1 expression. Furthermore, RARRES1 over expression elevates the levels of the antioxidant enzyme, catalase. Our results also indicate that RARRES1 expression inhibits angiogenesis in endothelial cells.

**Conclusions:**

In summary, the data presented here indicate that forced expression of RARRES1 in PCa cells (a) induces ER stress and autophagic response; (b) increases SIRT1 levels; and (c) higher levels of anti-oxidant enzymes. Our study also implicates the role of RARRES1 as a novel anti-angiogenic molecule. Overall this study reports the molecular players that RARRES1 modulates to serve as a tumor suppressor molecule. Future studies will help determine the *in vivo* mechanisms by which RARRES1 may serve as a target for therapeutic intervention both in cancer and in angiogenesis-related disorders.

## Introduction

Prostate cancer (PCa) is the most common cancer and a leading cause of cancer death among men in the United States. According to the American Cancer Society, 161,360 new cases and an estimated 26,730 deaths from PCa has been projected in 2017 alone[1]. Retinoic Acid Receptor Responder (RARRES1)/ Tazarotene-induced gene-1 (TIG-1) is a novel retinoid inducible gene first identified in skin raft cultures[2]. Frequent epigenetic inactivation due to aberrant DNA hypermethylation of the RARRES1 promoter results in transcriptional silencing of RARRES1 expression in esophageal, gastric, endometrial and PCa [3–7]. Cellular mechanisms via which RARRES1 exerts its tumor suppressor function have not been elucidated. The present study aims to understand the molecular mechanisms that underlie the functions of RARRES1.

Autophagy, an evolutionary conserved self-degradative process, plays the housekeeping role by eliminating damaged organelles and misfolded or aggregated proteins [8]. Three types of autophagy namely; macroautophagy, microautophagy, and chaperone-mediated autophagy have been reported. Nutrient deprivation, oxidative and endoplasmic reticulum stress, hypoxia, drug stimulation trigger the complex signaling mechanisms to induce autophagy[9]. Autophagy is a regulated multi-step process including nucleation, elongation, and autophagosome and autolysosome formation[10]. The role of autophagy in cancer is complex and its precise function remains a conundrum. Several reports indicate the dual role of autophagy: a tumor suppressor mechanism by preventing the accumulation of damaged organelles and misfolded aggregated proteins; and a tumor promoter function by facilitating cell survival mechanism for established tumors[11]. Defects in autophagy machinery have been associated with diverse pathological conditions including cancer, neurodegenerative, infectious, and metabolic diseases due to susceptibility to genomic instability and damage, and tumorigenesis indicating its tumor suppressor role[12]. On the contrary, autophagy also enables cell survival by maintaining energy production leading to tumor growth. Several studies suggest PI3K/Akt/mTOR signaling to be a critical pathway that negatively regulates autophagy and promotes cancer progression and is known to be upregulated in 30–50% of PCa. Furthermore, activation of ER stress response has been reported to induce autophagy by an orchestrated unfolded protein response (UPR). A highly conserved process, from yeast to mammals, autophagy helps regulate the cellular fate in case of various physiological and pathological conditions[13].

Angiogenesis, the formation of new blood vessels from pre-existing vessels, is a vital requirement for the growth and metastasis of tumors. It constitutes a pivotal step in cancer progression. For the tumors to grow and have the propensity to metastasize, they have to undergo the “angiogenic switch”[14]. This switch depends on the dynamic balance between the pro-angiogenic factors [such as hypoxia-inducible factor 1-alpha (HIF-1α) and vascular endothelial growth factor (VEGF)] and the anti-angiogenic factors [such as thrombospondin1 (TSP1), vascular endothelial growth inhibitor (VEGI)][15]. Stimuli for angiogenesis include hypoxia, inflammation or genetic variations in tumor suppressor and are a major hallmarks of cancer and several angiogenesis-related disorders[16].

The present study was performed to gain further insight into RARRES1 function. Our data indicate that RARRES1 over expression in PCa cell lines repress mitogen-activated protein kinase (MAPK) activation. RARRES1 expression was found to induce ER stress, autophagy and modulate mTOR and SIRT1 levels. Our results further demonstrate elevated levels of antioxidant enzymes, catalase and MnSOD upon RARRES1 expression. We also report here for the first time that RARRES1 expression inhibits angiogenesis. Together our study presents cellular mechanisms of RARRES1 function and highlights the important role it plays in modulating PCa growth and progression presenting a molecular target for therapeutic intervention.

## Materials and methods

### Cell lines and culture conditions

Human PCa cell lines, PC3 and C4-2, were obtained from Georgetown University (GU) Lombardi Comprehensive Cancer Center (LCCC) and cultured in Advanced RPMI medium (Invitrogen, Carlsbad, CA) supplemented with 5% (v/v) FBS and 2 mmol/L L-glutamine and antibiotic. HUVECs and the endothelial basal media were purchased from Lonza Ltd. All cells were maintained at 37°C in 5% CO_2_ and monitored for their typical morphology.

### RNA extraction, cDNA synthesis and Quantitative Real time (RT)-PCR

Total RNA was extracted using Trizol reagent (Invitrogen) as per manufacturer's instructions. 1 μg of total RNA was used for cDNA synthesis with random hexamers according to the High capacity cDNA Reverse transcription kit instructions (# 4368814, Applied Biosystems). The resulting cDNA was diluted 1:5 in nuclease-free water. Real time PCR primers were ordered from Integrated DNA Technologies. Reactions were performed in a 96-well fast reaction plate in ABI 7300, Real-Time PCR Detection System with 10 pM each of sense and antisense primer, and 2ul diluted cDNA. The SYBR green qPCR cycle was: 10 min incubation at 95°C followed by 40 cycles of amplification: 95°C for 15 sec and 60°C for 1 min. Reactions were run in triplicate in three independent experiments. Housekeeping gene GAPDH was used as an internal control to normalize the variability in expression levels. The relative RNA amount was calculated with the 2^-ΔΔCT^ method. Primers sequences[17] are as follows: Catalase For: TCCGGGATCTTTTTAACGC; Catalase Rev: TCGAGCACGGTAGGGACAG; MnSOD For: CTCCCCGACCTGCCCTACG; MnSOD-Rev: AAACCAAGCCAACCCCAAC; GPX For: GCGGCGGCCCAGTCGGTGTA; GPX Rev: GAGCTTGGGGTCGGTCATAA; HIF-1α For: AACATAAAGTCTGCAACATGGAAG; HIF-1α Rev: TTTGATGGGTGAGGAATGGG; TSP-1 For: CTCCCCTATGCTATCACAACG; TSP-1 Rev: AGGAACTGTGGCATTGGAG; Beclin For: AAGAGGTTGAGAAAGGCGAG; Beclin Rev: TGGGTTTTGATGGAATAGGAGC; IRE-1α For: GCGAACAGAATACACCATCAC; IRE-1α Rev: ACCAGCCCATCACCATTG; GRP78 For: GCTTTCACCATGTTTCCCAG; GRP78 Rev: GCTCACGCTATAATCCCAGTAC; GAPDH For:ACATCGCTCAGACACCATG; GAPDH Rev: TGTAGTTGAGGTCAATGAAGGG.

### p38MAPK activity assay

PC3 cells were transfected with empty vector or RARRES1 expression vector. The p38 MAPK activity was measured as per manufacturer’s protocol (Cat # 9820, Cell Signaling Technology, Denver, USA). Briefly, the cell lysate was immunoprecipitated with phospho-p38 MAPK immobilized beads overnight at 4°C. Upon centrifugation and washes, the pellet was incubated with substrate (ATF-2), kinase buffer and ATF2 for 30 min at 30°C. The reaction was terminated by adding 2x LDS buffer and was then heated at 100°C for 5 minutes before loading on SDS gel. The blot was immuno probed with phospho-ATF2 and total ATF2.

### Tube formation assay

The Matrigel assay was performed as per manufacturer’s instructions. Briefly, the matrigel was thawed and 50μl of Matrigel was added to flat bottom 96 well plate which was allowed to polymerize at 37°C for 1h. The HUVECs were then seeded on the matrigel after 48 hours of transfection with empty vector or RARRES1 expression vector at a density of 5 × 10^3^ cells per well at 37°C and 5% CO2 in endothelial basal growth complete media. After 18 hours of incubation, the tube-like structures were visualized and phase-contrast images were captured at 4X magnification using the Nikon microscope.

### SDS-PAGE and immunoblot analysis

PCa cells were lysed with ice cold 1X lysis buffer (Cell Signaling) supplemented with the protease phosphatase inhibitor cocktail (Roche). Protein was collected after 24 hours from cells transfected with RARRES1 expression vector or empty vector. Estimation of protein concentration was determined by Bradford reagent. Equal amounts (30 μg) of protein were loaded onto an acrylamide gel, separated by 4–12% PAGE, transferred to polyvinylidene difluoride PVDF membrane, and probed with primary antibodies; RARRES1 (1:1000, HPA003892 Sigma); Beclin (# 11427; Santa Cruz; SC). Also HA (#2367); phospho AMPK(#2535); phospho ERK (# 9101); phospho JNK (4668); phospho p38MAPK(# 9211); total JNK (#571, SC); total p38MAPK(#728); mTOR (#2983); SIRT1 (#2493); FOXO1 (# 2880); acetylated FOXO1 (#49437, SC); LC3B (#2775) and GAPDH (# 2118) were purchased from Cell Signaling technologies (Denver, MA).

### Immunofluorescence staining and confocal microscopy

Autophagy was detected by using confocal microscopy. Briefly, PCa cells were plated onto Lab TekII eight well chamber slides. PCa cells were transfected with RARRES1 expression vector or empty vector for 48 hours. The immunofluorescence assay was performed as described previously [18]. LC3B, HA were used as primary antibodies. The Alexa Fluor 488 goat anti-mouse antibody (A11034, Life technologies, MA) and the Alexa Fluor 594 goat anti-rabbit antibody (A11032, Life technologies, MA) were used as secondary antibodies. The coverslips were mounted with DAPI containing Vectashield (Vector Laboratories, H-1000). Samples were viewed with Zeiss 700 confocal microscope at indicated magnifications and analyzed using LSM software.

### Statistical analysis

All experiments were performed atleast three times and the data are presented as the ± SEM (indicated in the figure legends). Data were subjected to statistical analysis via Student *t* test. *P<0*.*05* was considered statistically significant.

## Results

### RARRES1 modulates PKC signaling

**Fig 1:**
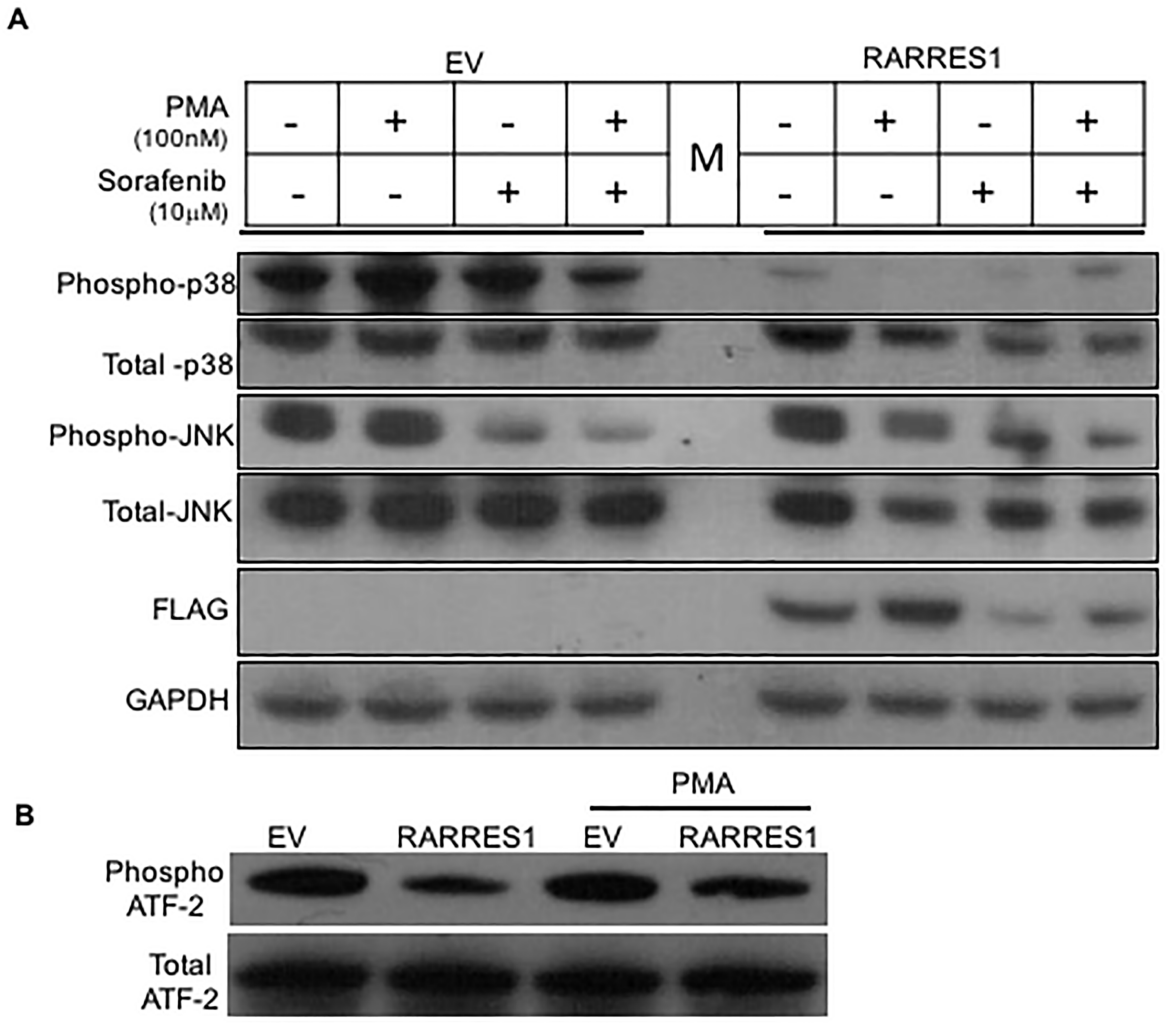
RARRES1 modulates protein kinase C (PKC) pathway. **A)** PC3 cells were induced by PKC agonist, phorbol myristate acetate (PMA) after transfection with empty vector or RARRES1 expression vector. Immunoblot analysis to detect the levels of PKC signaling pathway proteins on RARRES1 expression was performed with phospho antibodies specific against p44/42(ERK), p-p38MAPK, p-JNK and total ERK, p38MAPK and JNK along with GAPDH. Transfection efficiency of RARRES1 expression vector was determined by immunoblotting with FLAG antibody. **B)** PC3 cells were induced by PKC agonist, phorbol myristate acetate (PMA) after transfection with empty vector or RARRES1 expression vector. After 48 hours of transfection, cells were lysed and the whole cell lysate was immunoprecipitated with phospho-p38 MAPK antibody. The kinase reaction was performed with ATF-2 substrate and the bead-bound phospho-p38 Ab.

The MAP kinase signal transduction pathways play an important role in transducing extracellular signals to cellular responses and are involved in prostate cancer (PCa) development [19]. To determine if RARRES1 in PCa modulates the MAP kinase pathway, PCa cells were transfected with empty vector or RARRES1 expression vector. Our results indicate that RARRES1 inhibits the activation of the MAP kinase family members: extracellular signal-related kinase (ERK), Jun NH2 terminal kinase (JNK), and p38 MAPK. Furthermore, on treatment of PCa cells with phorbol 12-myristate 13-acetate (PMA), a PKC-MAP kinase inducer, our results indicate that RARRES1 modulates the expression of phosphorylated JNK and ERK. The expression levels of phosphorylated p38MAPK induced by PMA were significantly inhibited by RARRES1 expression (Fig 1A). To test if the kinase activity of p38 MAPK was altered by RARRES1 expression, *in vitro* phosphorylation of p38 MAPK substrate, ATF-2 was measured. Our results show that the levels of phosphorylated ATF-2 substrate are significantly lower in p38 MAPK isolated from the PCa cells expressing RARRES1 as compared to cells expressing empty vector(Fig 1B). The results indicate the role of RARRES1 in modulating p38 MAPK axis.

### RARRES1 induces autophagy

**Fig 2:**
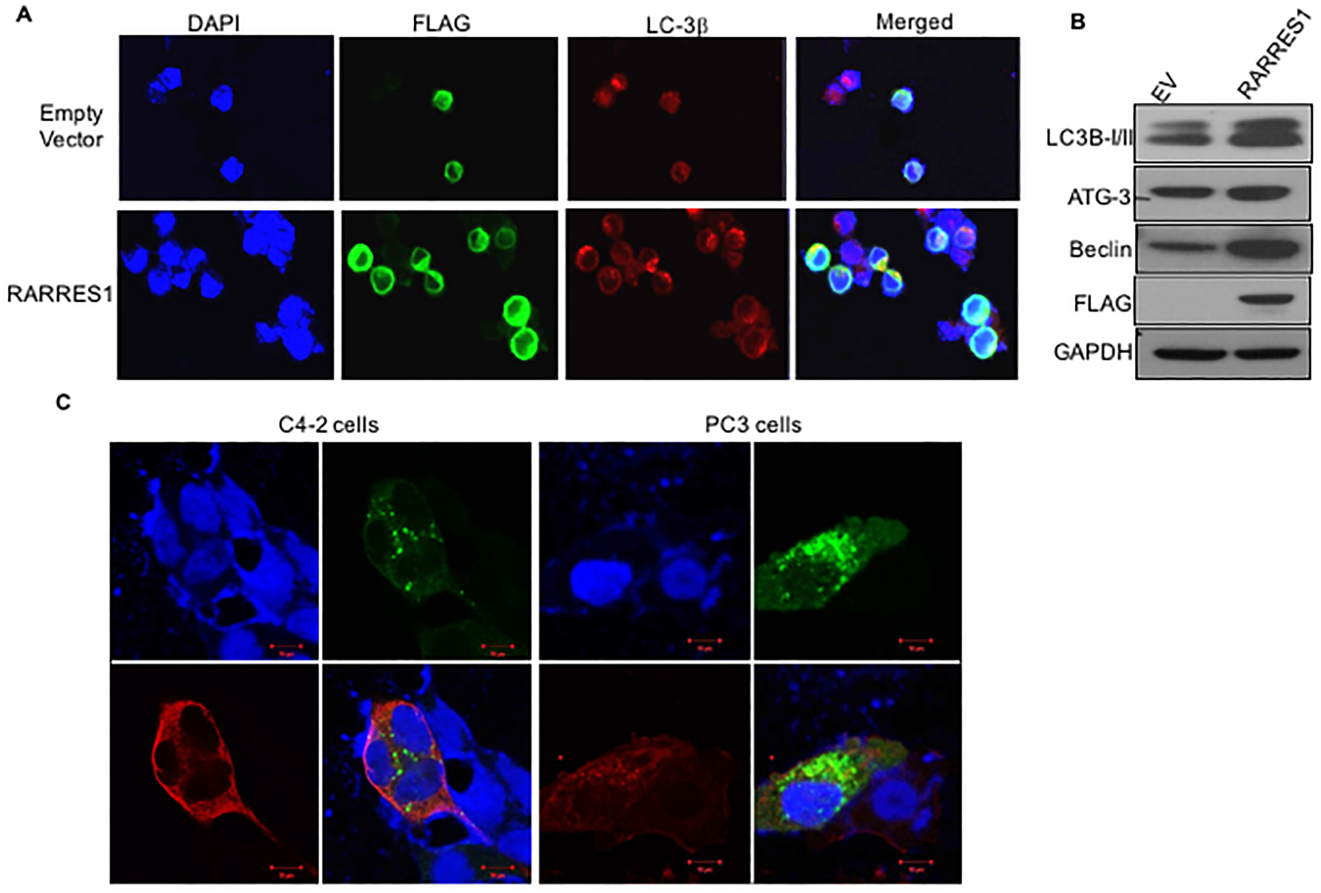
RARRES1 induces autophagy. **A)** PC3 cells were plated in 8 well chamber slides and transfected with empty vector or RARRES1 expression vector. Cells were then fixed with parafomaldehyde and immunocytochemistry with antibodies, FLAG and LC3B was performed. Confocal images were taken at 20X magnification. **B)** Protein lysates from PC3 cells transfected with empty vector or RARRES1 expression vector were immunoblotted and immunoprobed with antibodies against autophagy markers, LC3B, ATG3, Beclin, FLAG and GAPDH. **C)** Prostate cancer cells C4-2 and PC3 cells were co-transfected with LC3B and empty vector or RARRES1 expression vector. Immunocytochemistry was performed using LC3B (green) and FLAG (red) antibodies and images were taken with 40X objective using Zeiss LSM 700 confocal microscope.

Several reports suggest that the inhibition of p38MAPK triggers autophagy [20]. Since our data suggested a reduced kinase activity of p38MAPK, we sought to determine if autophagy is modulated by RARRES1. PC3 cells were transfected with empty vector or RARRES1 expression vector. Confocal images after immunocytochemistry with LC3B and HA tag antibodies showed an increased levels of LC3B punctuate bodies in cells expressing RARRES1 (Fig 2A). Expression levels of autophagy markers, beclin, ATG3 and LC3BII conversion from LC3BI were higher in PCa cells expressing RARRES1(Fig 2B). To determine if RARRES1 can cleave LC3B, we co-transfected C4-2 and PC3 cells with RARRES1 and LC3B expression vectors. Immunocytochemical images by confocal microscopy demonstrate that cells expressing the RARRES1 expression vector have more LC3B punctuate bodies than the cells that were not expressing RARRES1 indicating autophagic flux (Fig 2C).

### RARRES1 inhibits mTOR and induces SIRT1 and catalase

**Fig 3:**
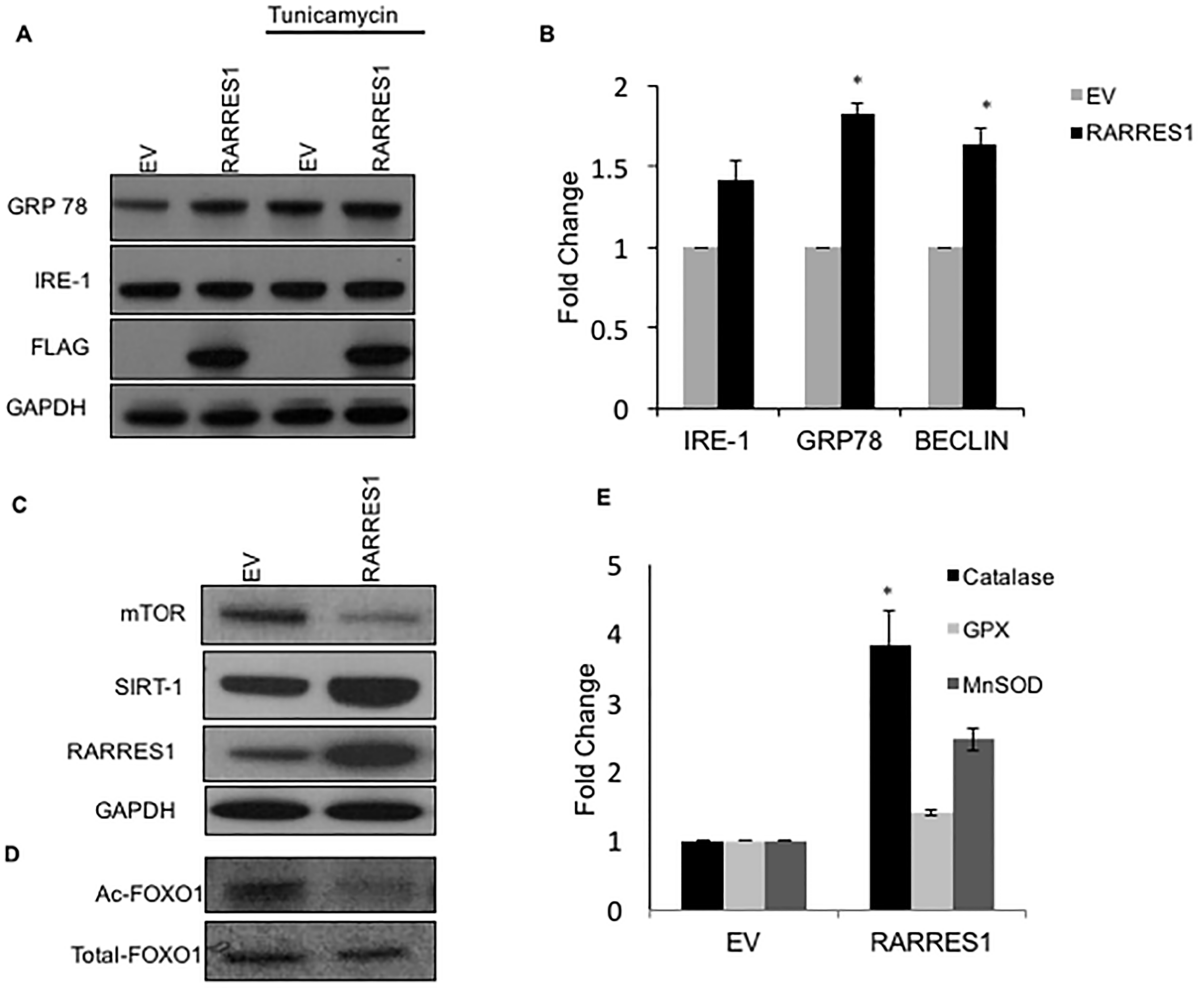
RARRES1 modulates ER stress and the expression of antioxidant enzymes. **A)** PC3 cells were treated with tunicamycin after being transfected with empty vector or RARRES1 expression vectors. Immunoblot analysis was performed for ER stress with antibodies against GRP78, IRE-1, FLAG and GAPDH (loading control). **B)** Real time qPCR data of PC3 cells transfected with empty vector or RARRES1 expression vector. Fold changes have been calculated by 2^^-(ΔΔCT)^ method; *p<0.05. **C)** PC3 cells were transfected with empty vector or RARRES1 expression vector. Immunoblot analysis was performed for autophagy modulating signaling proteins, with antibodies against mTOR, SIRT1, RARRES1 and GAPDH (loading control). **D)** Immunoblot data with antibodies against acetylated and total forms of FOXO1 with protein lysates from cells transfected with empty vector or RARRES1 expression vector. **E)** Levels of antioxidant enzymes, catalase, Glutathione peroxidase (GPX), Manganese superoxide dismutase (MnSOD) after empty vector or RARRES1 expression vector transfection; *p<0.01.

Having shown that RARRES1 expression induces autophagy, we wanted to identify the mechanism via which RARRES1 induces autophagy. Several studies report the important link between autophagic response and ER stress[21]. To determine if RARRES1 induces autophagy via the ER stress induction, we determined the levels of ER stress markers, GRP78/Bip and IRE-1 at RNA and protein levels. Tunicamycin, an ER stress inducer was used as a positive control. Our data shows increased levels of ER stress markers upon RARRES1 expression (Fig 3A and 3B). Since inhibition of mTOR is known to be a major “gatekeeper” in the process of autophagy induction[13, 22], we determined the levels of mTOR on RARRES1 expression. Our results indicate a significant repression of mTOR levels on RARRES1 expression (Fig 3C). Several studies report that SIRT1 is induced under nutrient deprivation and stress as sensed by NAD^+^/NADH ratio [23] and is also known to inhibit mTOR. To determine if RARRES1 expression could modulate the levels of SIRT1, the levels of SIRT1 expression were studied. Our result indicate that over expression of RARRES1 induces a significant increase in SIRT1 expression (Fig 3C). To determine whether over expression of SIRT1 by RARRES1 also modulates its activity we checked the acetylated levels of its target, forkhead box O1 (FOXO1). It is well known that SIRT1 deacetylates FOXO transcription factors [24]; FOXO1 thus can overcome the repression of acetylation on FOXO1 by cAMP-response element-binding protein (CREB)-binding protein[25]. Our results indicate that RARRES1 expression lowers the levels of acetylated FOXO1 as compared to the total levels of FOXO1. (Fig 3D). Since FOXO1 can modulate the synthesis of antioxidant enzymes [26] and it is well known that production of oxidative stress positively regulates tumor growth, we sought to determine if RARRES1 expression could modulate this phenomenon. Assay performed with dihydrorhodamine (DHR) 123 assay demonstrate that PCa cells when treated with hydrogen peroxide after RARRES1 transfection were unable to induce a significant production of reactive oxygen species when compared with empty vector transfected cells (data not shown). To understand this data we studied the levels of antioxidant enzymes, MnSOD, glutathione peroxidase and catalase upon RARRES1 transfection that may aid in quenching the production of reactive oxygen species. Our results show that PCa cells expressing RARRES1 vector have a significant induction of antioxidant enzyme, catalase when compared to its empty vector control (Fig 3E).

### RARRES1 expression inhibits *in vitro* angiogenesis

**Fig 4:**
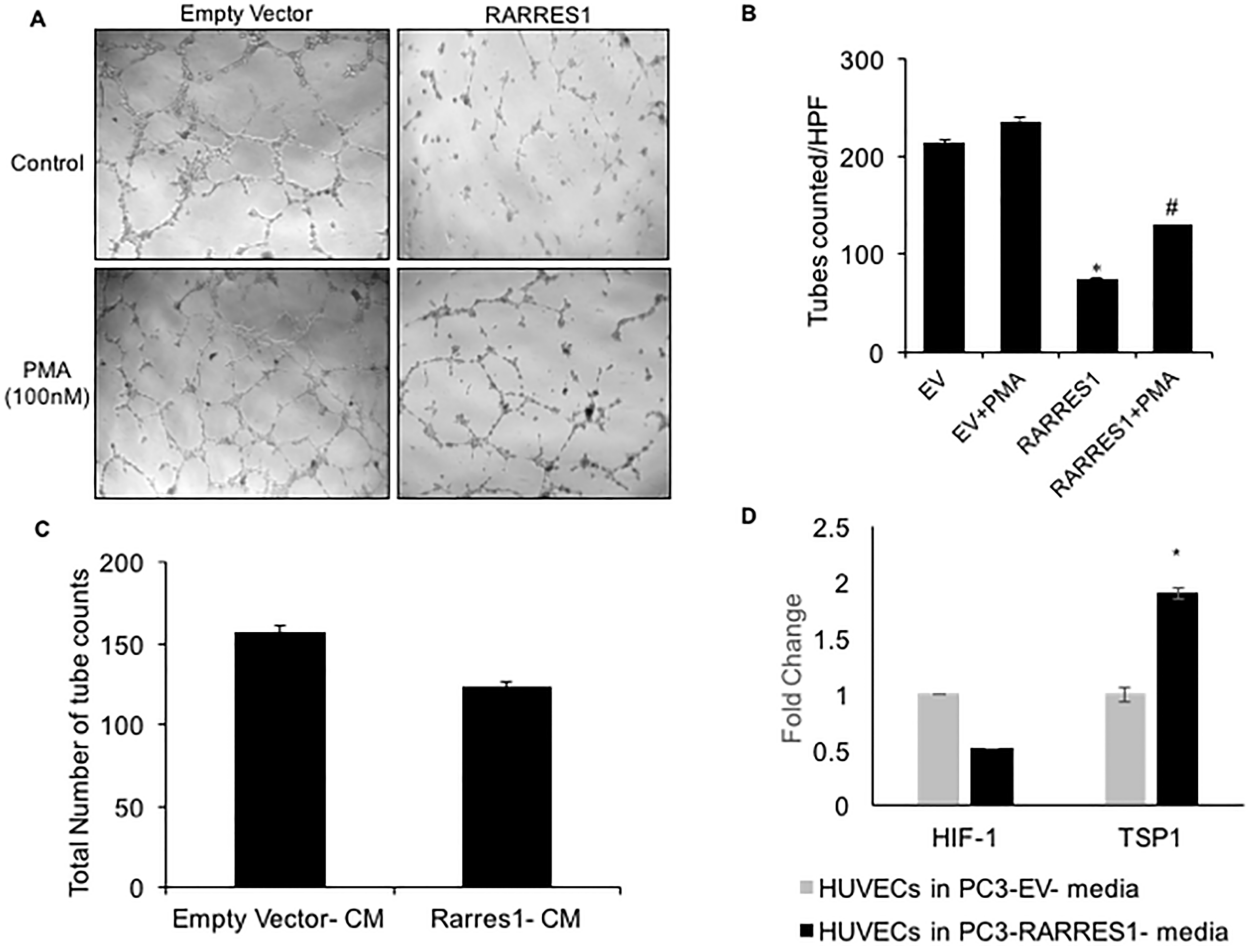
RARRES1 modulates angiogenesis. Human umbilical vein endothelial cells (HUVECs) were transfected with empty vector or RARRES1 expression vector. After 48 hours HUVECs were trypsinized and seeded on a matrigel at a density of 5×10^3^/well in triplicates. After 18h, tube-like structures were photographed. **A)** Representative photographs of tube-like structure. **B)** Quantitative analysis of total tube numbers; *p<0.01 vs. empty vector; #p <.05 vs. empty vector and PMA. **C)** PC3 cells were transfected with empty vector or RARRES1 expression vector. HUVECs were seeded on a matrigel at a density of 5×10^3^/well and treated with the conditioned media (CM) from PC3 transfected cells. Each group was in triplicate. After 18h, tube-like structures were photographed and quantitated. The quantitation results of total tube numbers are reported. **D)** Real time qPCR analysis of TSP1 and HIF1 expression on HUVECs treated with conditioned media from empty vector or RARRES1 expression vector transfected PC3 cells. Fold changes have been calculated by 2^^-(ΔΔCT)^ method, *p<0.05.

Accumulating evidence suggest that reactive oxidant species (ROS) may affect tumor promotion by mediating angiogenesis. Anti-oxidant therapies are being developed to target ROS-mediated angiogenesis. We hypothesized that the lower levels of ROS production and increased anti-oxidant levels on RARRES1 expression may modulate angiogenesis. To determine if RARRES1 expression could modulate angiogenesis, we transfected Human Umbilical Vein Endothelial Cells (HUVEC) with RARRES1 or empty vector as a control. The results demonstrate a significant inhibition of tube formation by RARRES1 expression (Fig 4A). Also, positive control, (PMA) could not alleviate this repression of angiogenic potential by RARRES1 expression (Fig 4B). Since signaling cues in and from cancer cells modulate angiogenesis *in vivo*, we placed HUVECs in matrigel supplemented with conditioned media (CM) from RARRES1-transfected PCa cells. Our results show difference in the total number of tube counts in the cells treated with CM from PC3 cells transfected with RARRES1 and empty vector(Fig 4C). The real time qPCR data also indicated a decrease in angiogenesis inducer, HIF-1α and significant increase in angiogenesis inhibitor, TSP1 in HUVECs treated with conditioned media from empty vector or RARRES1 transfected PC3 cells (Fig 4D).

## Discussion

Prostate cancer (PCa) is the most common malignancy in men and is the second leading cause of cancer deaths worldwide. Advances in the study of prostate carcinogenesis suggest a complex and multi-factorial role of epigenetic aberrations in the progression of the disease. The tumor suppressor gene, RARRES1, was reported to be hyper-methylated at its promoter region in several forms of cancer including PCa leading to suppression of RARRES1 [27]. We sought to determine the mechanism by which RARRES1 may function as a tumor suppressor in PCa.

Mitogen-activated protein kinase (MAPK) pathways are known to modulate cellular processes such as growth, proliferation, differentiation, migration and apoptosis. Deregulation of the MAPK signaling cascade play a critical role in cancer development and progression [28]. Several findings elucidate the oncogenic role of p38MAPK in triggering cancer related-processes, cell metabolism, invasion, inflammation and angiogenesis and its impact on the invasiveness of aggressive human PCa cells. p38 MAPKs are a family of serine/threonine-directed kinases commonly known as “stress-activated” kinases. Studies indicate that p38MAPK is a negative regulator of autophagy. Treatment with SB203580, a p38 specific inhibitor, on Sertoli cells *in vitro* shows accumulation of large autophagolysosomes [29]. p38MAPK has been reported to negatively modulate the autophagic process by phosphorylating Atg5, an E3 ubiquitin ligase that is required for autophagosome elongation and LC3B lipidation [30]. We report that RARRES1 expression inhibits the phosphorylated form of p38MAPK and also reduces its kinetic activity indicating possible mechanisms of tumor suppression and autophagy induction by RARRES1.

Where autophagy is found to be a process of self-digestion for survival under nutrient deprivation, it is also known to facilitate the removal of unfolded and damaged proteins and organelles[11]. Defects in autophagy have been associated with enhanced gene amplification and susceptibility to genomic damage and chromosomal instability, resulting in aneuploidy, a cancer hallmark. Mice deficient in autophagy related genes die in an early stage of embryogenesis [31]. Furthermore, in 40–75% of human breast, prostate, and ovarian cancers, monoallelic loss of Beclin1, an essential autophagy related gene has been reported, thereby suggesting a prime role of autophagy in tumor prevention [32]. Our results indicate that over-expression of RARRES1 induces autophagy related markers, Beclin1, ATG3 and the LC-3BII conversion from LC-3BI. To determine the mechanism of autophagy induction, we studied the level of well-known autophagy modulator, mTOR. An inhibition of mTOR by RARRES1 expression demonstrate that RARRES1 modulates autophagy and its inhibition in PCa cells may lead to cancer progression. Our data suggest that loss of RARRES1 could lead to increased levels of mTOR causing deficient autophagy suggesting and potential mechanism of PCa progression.

Sirtuin1 (SIRT1), a histone deacetylase has been reported to positively regulate autophagy [33] and thus has a multi-functional role in inflammatory diseases and cancer [34]. Studies also report that SIRT1 plays an important role in DNA damage repair and in maintaining genome stability. Homogenous SIRT1 embryos die at mid-gestation and displayed reduced chromosomal condensation along with impaired heterochromatin formation, and abnormal mitosis. Decreased SIRT1 levels have also been reported to cause reduced ability to repair DNA-double strand breaks (DSBs), and have impaired DNA Damage Response [35–36]. Furthermore, upon analyzing published SIRT1 expression database, *Wang et al*. reported that in human breast cancer, BRCA1 deficiency causes reduced SIRT1 levels and may be the cause for the malignant transformation[37–38]. Evidence also suggests that SIRT1 deacetylates the DNA methyltransferases (DNMTs) [39] that are known to add methyl groups to the 5′ position of cytosine residues of CpG dinucleotides thus interfering with the transcription factor binding thereby silencing the DNA [40]. Hypermethylation of DNA is known to promote chromosome instability and tumorigenesis[41–42] by silencing tumor suppressor genes. Our results demonstrating the elevated levels of SIRT1 upon RARRES1 overexpression suggest a possible feedback loop wherein RARRES1 modulates SIRT1 expression that may in turn prevent the methylation of RARRES1 promoter by deacetylating and inhibiting the activity of DNMT1. However, since RARRES1 levels are significantly low in PCa, SIRT1 cannot deacetylate DNMTs and thus RARRES1 promoter is predominantly hypermethylated as has been reported earlier[5]. It should be interesting to study the mechanism of SIRT1 induction by RARRES1 and if RARRES1-induced SIRT1 helps lower DSBs in PCa.

Angiogenesis is an important hallmark of tumor progression and our data showed that RARRES1 negatively modulates angiogenesis. SIRT1 is known to deacetylate and attenuate the transcription activity of FOXO1 [43], a positive regulator of anti-angiogenic protein, TSP1 [44]; inhibition of which leads to activation of angiogenesis-related genes, HIF-1α [45]. Our data implicates that RARRES1-induced SIRT1 results in significant increased deacetylation of FOXO1 protein, and higher levels of TSP1 along with reduced HIF-1 levels. Additionally, deacetylation of FOXO transcription factors by SIRT1 helps to serve as critical regulator of oxidative stress by modulating the expression of several anti-oxidant enzymes [26]. Oxidative stress mediated damage of cellular macromolecules have been implicated in several diseases including diabetes, aging and cancer [46]. Catalase, an antioxidant enzyme plays a crucial role in counteracting the production of ROS [47] and over expression of catalase was reported to impair the proliferation and migration capabilities of breast cancer cells, MCF-7 and increase sensitivity of cells to paclitaxel, etoposide and arsenic trioxide [48]. Further, mimetic of catalase, EUK 134 was reported to attenuate viability, proliferation, clonal expansion, adhesion, and migration of breast cancer cells, *in vitro* [49]. These evidence strengthen our findings of increased catalase production on RARRES1 expression, suggesting an alternative mechanism by which RARRES1 modulates oxidative stress, loss of which is detrimental in PCa.

**Fig 5:**
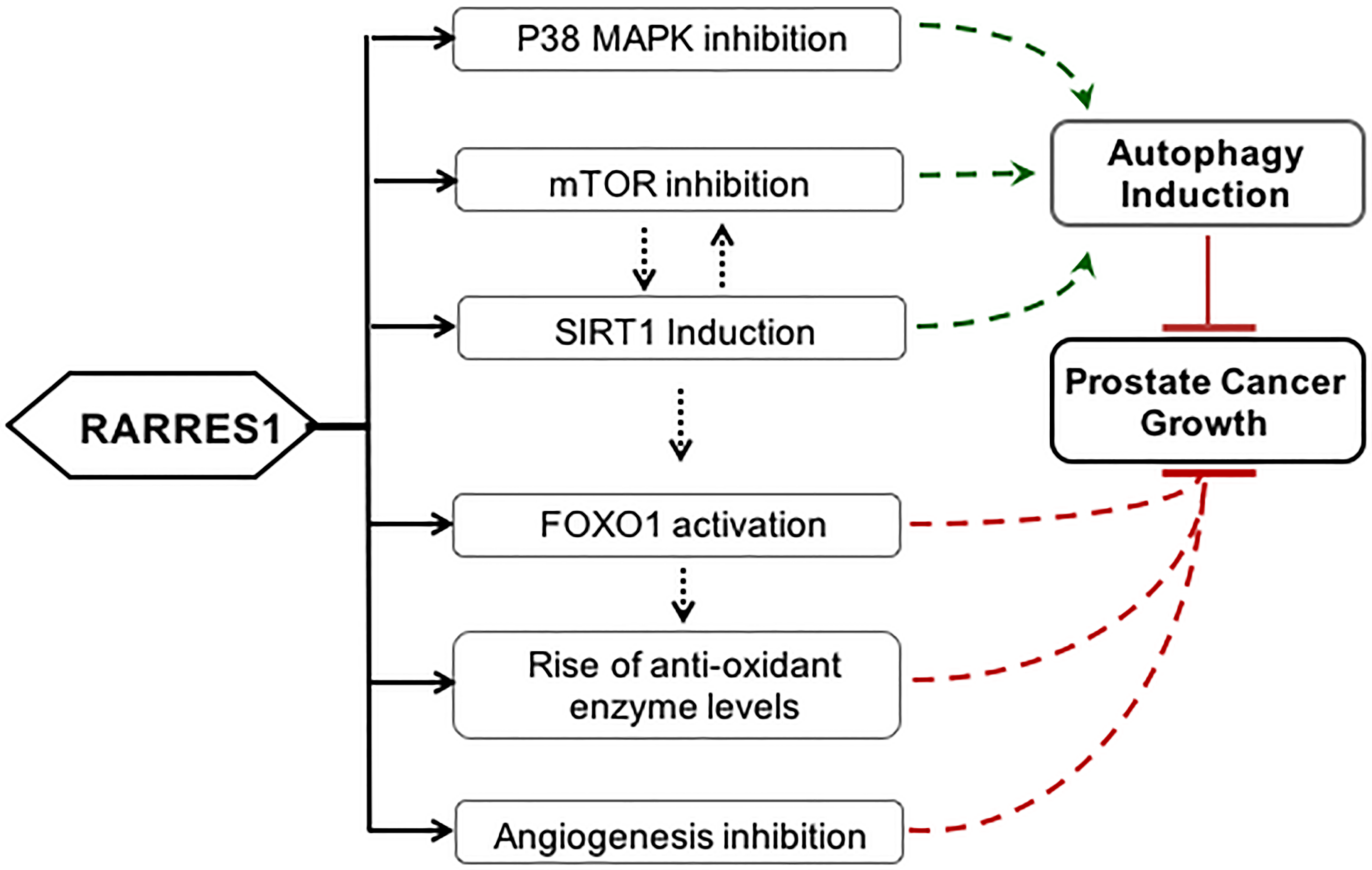
Mechanism of RARRES1 function. Based on our data and cited references, the outline of RARRES1’s role of tumor suppression in prostate cancer has been depicted. The green arrows indicate a positive induction and the red lines indicate repression. The black dashed arrows indicate the presence of intercellular signaling between the processes/molecules. The solid black arrows indicate the results we present in the paper to support our conclusion.

In conclusion, we have demonstrated that RARRES1 expression modulates a series of molecular signaling pathways inducing autophagy and inhibiting angiogenesis. Loss of RARRES1 in PCa can lead to deficient autophagy promoting genomic instability. Together the study provides insights into possible mechainsms of role of RARRES1 in PCa and how RARRES1 expression (Fig 5) may be of therapeutic benefit in PCa setting the stage for future *in vivo* investigations.

## Abbreviations

ER Stress: Endoplasmic Reticulum Stress
MAPK: Mitogen-activated protein kinase
mTOR: Mammalian target of rapamycin
ROS: Reactive oxygen species
PCa: Prostate cancer
RARRES1: Retinoic Acid Receptor Responder
SIRT1: Sirtuin 1
